# Two Novel Geminiviruses Identified in Bees (*Apis mellifera* and *Nomia* sp.)

**DOI:** 10.3390/v16040602

**Published:** 2024-04-13

**Authors:** Rohan Antonio Bandoo, Simona Kraberger, Arvind Varsani

**Affiliations:** 1 School of Life Sciences, Arizona State University, Tempe, AZ 85287, USA; 2 The Biodesign Center for Fundamental and Applied Microbiomics, Arizona State University, Tempe, AZ 85287, USA; 3 Center for Evolution and Medicine, Arizona State University, Tempe, AZ 85287, USA; 4 Structural Biology Research Unit, Department of Integrative Biomedical Sciences, University of Cape Town, Rondebosch, Cape Town 7700, South Africa

**Keywords:** *Geminiviridae*, honeybee, solitary bee, *Apis mellifera*, *Nomia* sp.

## Abstract

Members of the *Geminviridae* family are circular single-stranded DNA plant-infecting viruses, some of which impact global food production. Geminiviruses are vectored by sap-feeding insects such as leafhoppers, treehoppers, aphids, and whiteflies. Additionally, geminivirus sequences have also been identified in other insects such as dragonflies, mosquitoes, and stingless bees. As part of a viral metagenomics study on honeybees and solitary bees (*Nomia* sp.), two geminivirus genomes were identified. These represent a novel citlodavirus (from honeybees collected from Westmoreland, Jamaica) and a mastrevirus-like genome (from a solitary bee collected from Tempe, Arizona, USA). The novel honeybee-derived citlodavirus genome shares ~61 to 69% genome-wide nucleotide pairwise identity with other citlodavirus genome sequences and is most closely related to the passion fruit chlorotic mottle virus identified in Brazil. Whereas the novel solitary bee-derived mastrevirus-like genome shares ~55 to 61% genome-wide nucleotide identity with other mastreviruses and is most closely related to tobacco yellow dwarf virus identified in Australia, based on pairwise identity scores of the full genome, replication-associated protein, and capsid protein sequences. Previously, two geminiviruses in the *Begomovirus* genus were identified in samples of stingless bee (*Trigona* spp.) samples. Here, we identify viruses that represent two new species of geminiviruses from a honeybee and a solitary bee, which continues to demonstrate that plant pollinators can be utilized for the identification of plant-infecting DNA viruses in ecosystems.

## 1. Introduction

*Geminiviridae* is a family of plant-infecting circular single-stranded DNA (ssDNA) viruses in the phylum *Cressdnaviricota* [1]. Geminiviruses infect both monocotyledonous and dicotyledonous plants and many cause disease in agriculturally important plants. They are vectored by insects, namely, aphids, leafhoppers, treehoppers, and whiteflies [2,3,4,5,6]. Additionally, geminiviruses have been identified in insect predators such as dragonflies [7], as well as pollinators such as mosquitoes [8] and stingless bees [9,10].

Geminiviruses have small geminate virions (~22 × 35 nm) with mono- or bipartite circular ssDNA genomes approximately between 2.5 and 5.2 kb in size [2,11,12]. To date, there are 14 established genera and over 500 species within the family *Geminiviridae* [2]. The genera are *Becurtovirus*, *Begomovirus*, *Capulavirus*, *Citlodavirus*, *Curtovirus*, *Eragrovirus*, *Grablovirus*, *Maldovirus*, *Mastrevirus*, *Mulcrilevirus*, *Opunvirus*, *Topilevirus*, *Topocuvirus*, and *Turncurtovirus* [2]. Geminivirus genomes have between four and nine open reading frames (ORFs), and all encode a capsid protein (CP), replication-associated protein (Rep), and movement protein (MP). The Rep is the most conserved with three primary domains; HUH-endonuclease [13], Superfamily 3 (SF3) helicase [14], and Gemini Rep Sequence (GRS) domain motif [15].

Members of the *Citlodavirus* genus have thus far been detected in camellia [16], Chinese bayberries [17], passion fruit [18], paper mulberries [19], and citrus plants [20,21,22]. It is postulated that citlodaviruses are transmitted by whitefly vectors such as *Parabemisia myricae,* which thrives on wooded plants [22]. The CPs of citlodaviruses are most closely related to those of begomoviruses, which are vectored by the whiteflies *Bemisia tabaci*; however, the vector of citlodaviruses is yet to be determined [18]. Citlodaviruses have monopartite genomes that are ~12 to 30% larger than other monopartite geminiviruses and encode six genes. These genes on the complementary sense strand are the *rep* and *repA* (which code for Rep and RepA), and on the virion sense strand are the *cp* (codes for CP), *mp* (codes for MP), and two other genes, *v2* and *v3* (coding for V2 and V3 proteins, whose function is unknown). The MP is similar in size to those of bipartite begomovirus genomes [18]. Species within the *Citlodavirus* genus are determined based on a genome-wide pairwise identity threshold of 78%, and there are currently five established species within the genus *Citlodavirus* [23].

Members of the *Mastrevirus* genus have monopartite genomes ranging in size between 2.6 and 2.8 kb. They are transmitted by leafhoppers in the family Cicadellidae [24,25,26,27]. Mastreviruses are categorized as monocot-infecting or dicot-infecting, based on the host plant species they infect, and these two groups of mastreviruses are phylogenetically distinct [28]. The *rep* and *repA* genes are encoded on the complementary sense strand, whereas the *cp* and *mp* genes are encoded in the virion sense strand. The species demarcation threshold for mastreviruses is 78% genome-wide pairwise identity [28], and there are currently 45 established mastrevirus species [2].

Honeybees (*Apis mellifera*, family Apidae) and solitary bees (*Nomia* sp. family Halictidae) are associated with the pollination of plants as they forage for nectar and pollen across numerous flowering plants. Thus, bees can be useful surveillance tools for plant-infecting viruses, especially those that are pollen-associated [29,30,31]. Plant-infecting RNA viruses in the families *Alphaflexiviridae*, *Amalgaviridae*, *Aspiviridae*, *Betaflexiviridae*, *Bromoviridae*, *Chrysoviridae*, *Luteoviridae*, *Mayoviridae*, *Partitiviridae*, *Phenuiviridae*, *Potyviridae*, *Secoviridae*, *Solemoviridae*, *Tombusviridae*, *Tymoviridae,* and *Virgaviridae* have been found in honeybees or in pollen on honeybees [31,32,33,34,35,36,37,38,39,40,41,42]. On the other hand, little is known about plant-infecting DNA viruses in honeybees and only two reports have identified geminiviruses (genus *begomovirus*) in stingless bees (*Trigona* spp.) in Mexico [9,10].

As part of a broader viral metagenomic study of viruses in bees, here we used a viral metagenomics approach to identify two geminiviruses, a citlodavirus genome in a honeybee (*Apis mellifera*) from Jamaica and a mastrevirus-like genome in a solitary bee (*Nomia* sp.) from Tempe, Arizona, USA.

## 2. Materials and Methods

### 2.1. Sample Collection

Sampling of honeybees in Westmoreland, Jamaica, was undertaken at two local apiaries in March 2023. Twenty adult honeybees were collected from the two apiaries and frozen overnight before being air-dried at room temperature. Dried honeybee samples were transferred to the USA with prior approval of the Jamaican Ministry of Agriculture and Fisheries as well as the United States Department of Agriculture/APHIS. Additionally, 20 solitary bees (*Nomia* sp.) were collected in Tempe, Arizona (USA) in May 2023. All bees were stored at −80 °C until further processing.

Each of the samples was homogenized in 1 mL of SM Buffer (0.1 M NaCl, 10 mM MgSO_4_, 50 mM Tris-HCl [pH 7.4]). Homogenates were centrifuged at 9000 rpm for 1 min, and 200 µL the supernatant was used to isolate nucleic acid with the High Pure Viral Nucleic Acid kit (Roche Diagnostics, Indianapolis, IN, USA).

### 2.2. High-Throughput Sequencing, De Novo Assembly, and Identification of Viral-like Contigs

An aliquot of the viral DNA from the 20 individual honeybee samples and 20 solitary bee samples from each location was pooled and this was then used for rolling circle amplification (RCA) reaction with TempliPhi 2000 kit (Cytiva Lifesciences, Marlborough, MA, USA). RCA products were then combined with the viral nucleic acid at a 1:1 ratio and used to generate Illumina sequencing libraries using the Illumina DNA library prep kit (Illumina, San Diego, CA, USA) and sequenced on an Illumina NovaSeq X plus sequencer (Illumina, San Diego, CA, USA) sequencing platform at Psomagen Inc. (Rockville, MD, USA).

Raw reads (2 × 150 bp) were quality-checked and trimmed with Trimmomatic v0.39 [43]. Trimmed reads were de novo assembled using MEGAHIT v1.2.0 [44]. Assembled contigs > 1000 nt were analyzed against a viral RefSeq protein database (Release 220) using DIAMOND BLAST [45]. The viral-like contigs were determined to be circular if they had terminal redundancy. All viral-like contigs were annotated using Cenote-taker 2 [46]. The open reading frame identification was refined in Geneious Prime 2023.2.1 (Dotmatics, Boston, MA, USA).

### 2.3. Screening and Verification of Geminivirus Genomes

Based on the sequences of the two geminiviruses, we designed two sets of screening primers that target ~1060 nt of the *rep* gene. The RCA products of the 20 honeybees were individually screened for the citlodavirus sequence using the primer pair screen-acitlo (5′-GTC AAC GTT GAT GTC CCA CCA CTG-3′/R: 5′-CCT CAC TGC CAA AAA CAT CTT CCT CAC TTA CTC-3′) and those of the 20 solitary bees were screened for the mastrevirus-like sequence using the primer pair screen-nmast (5′-CAT ACA GAA AGA CGA TGG TAC AAT TGG CTT C-3′/R: 5′ CTT CTA ATC GTA GCA GAG CTT TCAG AAT CTG TGC-3′). The following PCR protocol using HotStart HiFi DNA polymerase (Roche Diagnostics, Indianapolis, IN, USA) and the specific primer pair was used for the amplification: 98 °C for 2 min for initial denaturation followed by 25 cycles of 98 °C for 30 s, 60 °C for 30 s, 72 °C for 1 min, and then a final 72 °C for 2 min prior to renaturation at 4 °C for 10 min. The amplicons were resolved in a 0.7% agarose gel.

To amplify the full genomes from positive screened samples, we designed sets of abutting primers, i.e., full-acitlo 5′-GCT GAG TAA ATG TAG GAT GAC TGT TGA TAG-3′/5′-GTG ACT TTT ATC AGG CTG TAA GTT AGG TAG-3′ for the citlodavirus genome and full-nmast 5′-CCT TTA TTC CTT GAA TAA ATT CTT CCG GCG-3′/5′-GAC GTG AAG AGT ACT CAT AAG GAT ATA CCT-3′ for the mastrevirus-like genome. The PCR amplifications were carried out using Kapa HotStart HiFi DNA polymerase (Roche Diagnostics, Indianapolis, IN, USA) with the specific primer pair, and the following thermal cycling conditions were used for the amplification of the citlodavirus genome, 98 °C for 2 min for initial denaturation followed by 25 cycles of 98 °C for 30 s, 65 °C for 30 s, 72 °C for 4 min, and then a final 72 °C for 5 min prior to renaturation at 4 °C for 10 min. For the mastrevirus-like genome PCR amplification, the specific primer pair and the following thermocycling condition were used for the amplification of the virus genome, 98 °C for 2 min for initial denaturation followed by 25 cycles of 98 °C for 30 s, 55 °C for 30 s, 72 °C for 2.5 min, and then a final 72 °C for 3 min prior to renaturation at 4 °C for 10 min. The PCR amplicons were resolved in a 0.7% agarose gel and amplicons of the ~2.5 and 4 kb were excised from the gel, purified, and cloned into pJET 1.2 vector (Thermo Fisher Scientific, Waltham, MA, USA) and then transformed into competent XL blue *E. coli* cells. The *E. coli* transformants were screened by PCR to confirm plasmids with the correct inserts. The recombinant plasmids were purified using the Fast-DNA spin plasmid purification kit (iNTRON technologies, Seongnam, Republic of Korea) and Sanger sequenced at Macrogen Inc. (Seoul, Republic of Korea) by primer walking. The Sanger sequence reads of the geminivirus genomes were assembled and annotated using Geneious Prime 2023.2.1 (Dotmatics, Boston, MA, USA).

### 2.4. Geminivirus Sequence Analyses

Representative geminivirus genomes (representing each species) were downloaded from GenBank and linearized at the nonanucleotide sequence. In the case of begomoviruses, we subsampled the species dataset with a representative with an 80% genome-wide pairwise identity.

The genomes were aligned using MAFFT [47] and a Neighbor-joining phylogenetic tree was inferred with Jukes Cantor substitution model and 1000 bootstrap iterations.

From this representative genome dataset, the *rep* and *cp* gene sequences were extracted, translated, and used to assemble Rep and CP amino acid sequence datasets that were aligned using MAFFT [47]. Amino acid substitution models RtRev+G+F for Rep and LG+G+F for CP were determined as the best-fit models using ProtTest 3 (Darriba et al. 2011) and these were used to infer maximum likelihood Rep and CP amino acid sequence phylogenetic trees using PhyML3 [48]. TreeGraph2 [49] was used to collapse branches with <0.8 aLRT support. The phylogenetic trees were rooted with genomovirus Rep sequences for the Rep amino acid phylogeny and satellite tobacco necrosis virus CPs for the CP phylogeny as outgroups. The phylogenetic trees were visualized in MEGA V11 [50]. All pairwise identity comparisons (nucleotide and amino acid) were undertaken using SDT v1.2 [51]

## 3. Results

### 3.1. Identification of Geminiviruses

In the de novo assembled contigs from the pools of the 20 honeybees from Jamaica and 20 solitary bees from Tempe, Arizona (USA), we identified two contigs of 3918 and 2541 nt that had terminal redundancy and high similarities to geminiviruses. One of these (3918 nt) from a honeybee in Westmoreland, Jamaica, is most similar to passion fruit chlorotic mottle virus (MG696802) in the *Citlodavirus* genus with 73.19% identity, 71% genome coverage, and e-value of zero, based on BLASTn analysis. The second contigs of 2541 nt from solitary bees in Tempe, Arizona, USA, has the top BLASTn hit to chickpea chlorotic dwarf virus (MN178605) in the *Mastrevirus* genus with 71.77% identity, 17% genome coverage, and an e-value of 4 × 10^−34^.

### 3.2. Citlodavirus Genome Identified in a Honeybee from Jamaica

**Figure 1:**
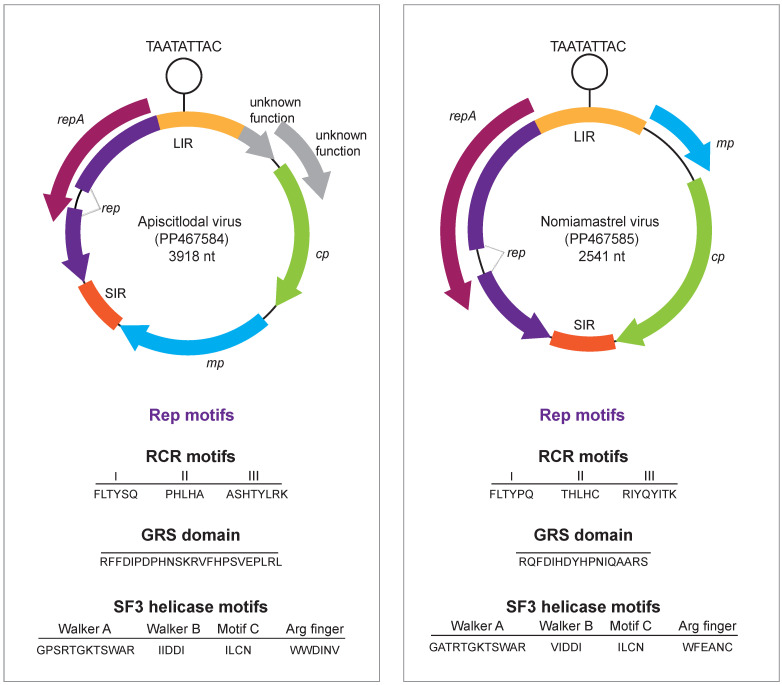
Illustration of the genome organization of apiscitlodal virus and nomiamastrel virus and a summary of the RCR and SF3 motifs identified in their Rep sequences.

We screened the 20 individual bee samples from those collected in Westmoreland, Jamaica, using the primer pair screen-acitlo and identified the citlodavirus genome in one sample. From this sample using the abutting primer pair full-acitlo, the full genome was amplified, cloned, and Sanger-sequenced. This virus was named the apiscitlodal virus, with the name being derived from *Apis* and citlodavirus. The genome was deposited in GenBank under accession number PP467584. Within this genome (3918 nt), we identified the nonanucleotide motif TAATATTAC, which is relatively conserved across most geminiviruses and all identified citlodaviruses. Within the genome, we identified two ORFs of unknown function (*v2*, 375 nt; *v3*, 231 nt), *cp* (744 nt), and *mp* (888 nt) genes in the virion sense strand. In the complementary sense strand, we identified the *repA* (864 nt) gene and spliced *rep* gene (1074 nt) with a 99 nt intron (Figure 1). Within the Rep sequence, we identified the conserved RCR motifs I (FLTYSQ), II (PHLHA), and III (ASHTYLRK), and the GRS domain (RFFDIPDPHNSKRVFHPSVEPLRS) and the SF3 helicase motifs: Walker A (GPSRTGKTSWAR), Walker B (IIDDI), and Motif C (VLCN). The arginine finger domain (WWDINV) was also present (Figure 1).

**Figure 2:**
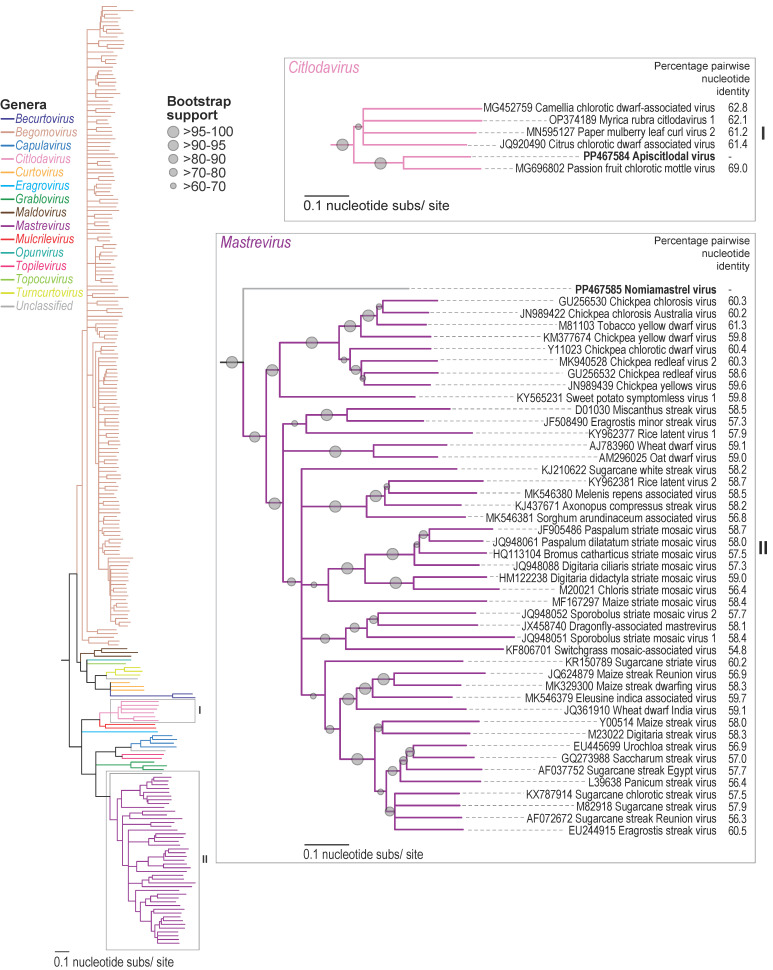
A neighbor-joining phylogenetic tree of representative genomes of geminiviruses, and the apiscitlodal virus and nomiamastrel virus. A detailed view of the phylogeny is provided in panel I for the citlodaviruses and panel II for the mastreviruses. Pairwise identities relative to the genome of apiscitlodal virus and nomiamastrel virus sequences are provided in panel I and II.

**Figure 3:**
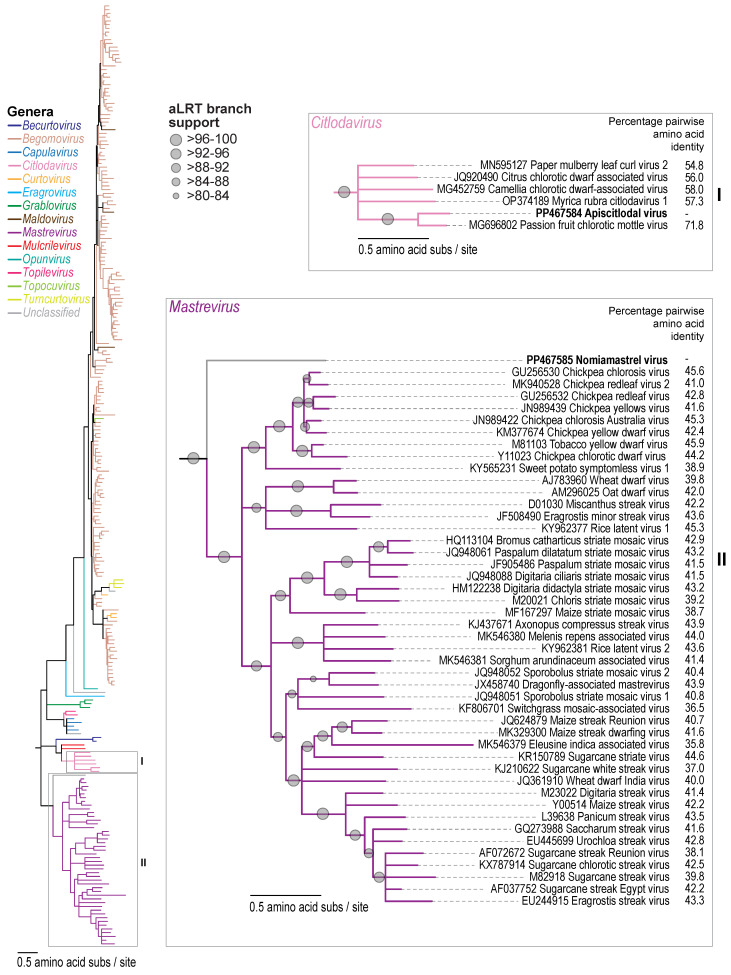
A maximum-likelihood phylogenetic tree of the Rep amino acid sequences of representative geminiviruses showing the phylogenetic relationship of apiscitlodal virus and nomiamastrel virus sequences. A detailed view of the phylogeny is provided in panel I for the citlodaviruses and panel II for the mastreviruses. Pairwise identities relative to the Reps of apiscitlodal virus and nomiamastrel virus sequences are provided in panels I and II.

**Figure 4:**
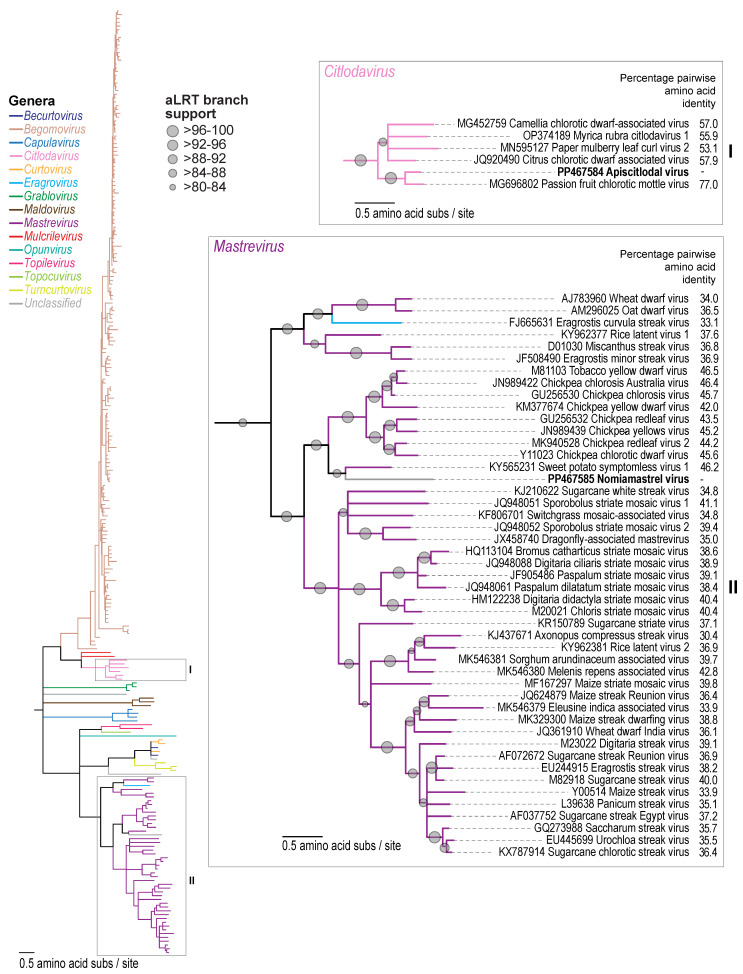
A maximum-likelihood phylogenetic tree of the CP amino acid sequences of representative geminiviruses showing the phylogenetic relationship of apiscitlodal virus and nomiamastrel virus sequences. A detailed view of the phylogeny is provided in panel I for the citlodaviruses and panel II for the mastreviruses. The CP amino acid sequence of eragrosvirus [54] is most closely related to that of mastreviruses and, thus, is included in panel II with those of mastreviruses. Pairwise identities relative to the CP amino acid sequences of apiscitlodal virus and nomiamastrel virus sequences are provided in panels I and II.

Similar to members of *Citlodavirus,* the apiscitlodal virus has a relatively large genome (3918 nt) and large *mp* gene (888 nt) (Figure 1). The larger genomes of citlodaviruses (~12 to 30%) have been attributed, at least in part, to a larger *mp* gene (~891 to 921 nt) than those identified in monopartite genomes of viruses in other geminivirus genera [18]. The larger *mp* gene of citlodavirus is most similar in size to the *mp* gene in the DNA-B molecule of bipartite begomoviruses (~714 to 1107 nt) [18]. Furthermore, while the full genome sequences of citlodaviruses are phylogenetically more closely related to the sequences of mulcrileviruses [19,52,53], the Rep amino acid sequences of both genera are more closely related to those of becurtoviruses, while their CP amino acid sequences are most similar to those of begomoviruses (Figure 2, Figure 3 and Figure 4). Based on these observations, it is postulated that members of *Citlodavirus* may represent intermediates between becurtovirus monopartite geminivirus genomes and bipartite begomovirus genomes (Fontenele et al. 2018a) [18].

Genome-wide pairwise analysis of the representative genomes of citlodaviruses showed that the apiscitlodal virus shares 61–69% identity, with the highest identity with the passion fruit chlorotic mottle virus (MG696802) [18] (Figure 2). Based on the genome-wide species demarcation threshold of 78% for citlodaviruses [23], the apiscitlodal virus represents a new species in the genus *Citlodavirus*.

Pairwise analysis of the V2, V3, CP, MP, and Rep amino sequences (Supplementary Data S1) of the apiscitlodal virus shows that they share the highest identities of 47.1%, 45.3%, 77.0%, 71.0%, and 71.8% to those of the passion fruit chlorotic mottle virus (MG696802). Phylogenetic analyses of Rep and CP amino acid sequences show that they are most closely related to those of the passion fruit chlorotic mottle virus within the *Citlodavirus* genus (Figure 3 and Figure 4).

### 3.3. Mastrevirus-like Genome Identified in a Solitary Bee from Arizona, USA

From one of the 20 solitary bee samples collected in Tempe, Arizona, USA, a mastrevirus-like genome (2541 nt) was recovered. We named this virus the nomiamastrel virus, with the name derived from *Nomia* and mastrevirus-like. This genome was deposited in GenBank under accession number PP467585. The nonanucleotide motif TAATATTAC was identified within the stem loop in the long intergenic region. This nonanucleotide motif is relatively conserved with 9 species of 47 in the genus showing variations to this sequence. In the genome of the nomiamastrel virus, we identified the *mp* (285 nt) and *cp* (804 nt) genes in the virion sense strand. The *repA* gene (867 nt) and a spliced *rep* gene (999 nt) with a 99 nt intron were identified in the complementary sense strand (Figure 1). The RCR motifs I (FLTYPQ), II (THLHC), and III (RIYQYITK), and the GRS domain (RQFDIHDYHPNIQAARS) and SF3 helicase motifs Walker A (GATRTGKTSWAR), Walker B (VIDDI), and Motif C (ILCN), as well as the Arginine finger domain (WFEANC), were identified in the Rep amino acid sequence (Figure 1).

The genome of the nomiamastrel virus shares ~55 to 61% genome-wide pairwise nucleotide identity with other representative mastreviruses (Figure 2), sharing the highest identity (61.3%) with the genome of the tobacco yellow dwarf virus (M81103) [55], a dicot-infecting mastrevirus. Similar to citlodaviruses, the genome-wide species demarcation threshold of mastreviruses is 78% [28]. Based on these criteria, the nomiamastrel virus represents a new species.

The CP and Rep amino acid sequences of the nomiamastrel virus share the highest pairwise identity of 46.5% and 45.9% (Figure 3 and Figure 4; Supplementary Data S2), respectively, with those of the tobacco yellow dwarf virus (M81103). The MP, on the other hand, shares the highest amino acid pairwise identity of 36.3% with that of the wheat dwarf virus (AJ783960) [56]. This virus has been labeled as a “mastre-like” virus due to its phylogenetic relationship of the full genome and Rep protein sequences that place it basal to all other mastreviruses (Figure 3). However, the CP amino acid sequence phylogenetic tree (Figure 4) shows that the nomiamastrel virus sits in a clade with the sweet potato symptomless virus (KY565231) [57,58], which is basal to those of the other dicot-infecting mastreviruses. Therefore, it is unclear whether this is a true mastrevirus or represents a related geminivirus. The CP amino acid sequence phylogeny of geminiviruses has previously been shown to be congruent with the evolutionary relationship with the insect vector that transmits them [59], the dicot-infecting mastreviruses vector identified as leafhoppers in the *Orsius* genus. Although the vector for the sweet potato symptomless virus is yet to be confirmed, it is probable that leafhoppers are the vector of both this virus and the nomiamastrel virus given the relationship between these CP amino acid sequences and those from the other dicot-infecting mastreviruses. To our knowledge, leafhoppers in the *Orsius* genus [60] have not been documented in North America; however, species belonging to other genera, such as the potato leafhopper (*Empoasca fabae*) and threecornered alfalfa hopper (*Spissistilus festinus*), have been identified in Arizona. Further studies are needed to elucidate the vector and plant host range of the nomiamastrel virus.

## 4. Discussion

Plant-infecting viruses are a serious threat to food security globally. While in their infancy, approaches that seek to indirectly sample viruses that likely infect plants via the sampling of common pollinators have been investigated [9,10,31,32,33,34,35,36,37,38,39,40,41,42,61]. This effort has mainly focused on plant-infecting RNA viruses using targeted and non-targeted assays. Nonetheless, two plant-infecting geminiviruses in the genus *Begomovirus* have been identified in stingless bees using targeted PCR assays [9,10]. Plant-infecting DNA viruses have also been identified in female mosquitos that feed on nectar [8] and dragonflies that feed on plant-infecting virus vectors [7]. Although geminiviruses have been identified in insect vectors, they are plant-infecting viruses.

Here, we report the identification of two geminiviruses (apiscitlodal virus and nomiamastrel virus) that represent two new species, one in the *Citlodavirus* genus, and the other putatively *Mastrevirus* (albeit a diverse one) from a honeybee (sampled in Jamaica) and a solitary bee (sampled in Arizona, USA), respectively. These two viruses are clearly plant-infecting viruses based on their genome organization as well as the relationship of their proteins with those of other geminiviruses (Figure 1, Figure 2, Figure 3 and Figure 4 and Supplementary Data S1 and S2). Nonetheless, the vectors as well as the plant host range of these viruses are unknown and need to be investigated in the future.

Jamaica hosts a diversity of cultivated and non-cultivated plants [62,63] and pollinators [64,65]. No information on viruses circulating amongst bees in Jamaica is available. However, plant-infecting viruses in the families *Bromoviridae*, *Caulimoviridae*, *Closteroviridae*, *Endornaviridae*, *Geminiviridae*, *Potyviridae*, *Secoviridae,* and *Solemoviridae* based on sequence information in NCBI Virus [66] have been reported. The identification of the citlodavirus in a honeybee suggests that the apiscitlodal virus adds to this list and is likely circulating in plants in at least the Westmoreland region of Jamaica. Begomoviruses have been identified in non-cultivated and cultivated crops in Jamaica in the past [67,68,69,70,71], but this is the first report of a citlodavirus. More broadly, citlodaviruses so far have only been identified in cultivated plants from Brazil, China, Thailand, and Turkey infecting camellia, Chinese bayberry, paper mulberry, passion fruit, and various citrus plants.

In contrast to the tropical Caribbean Island of Jamaica, Arizona in the southwestern USA has a more arid climate. Several crops are farmed in Arizona, with alfalfa, cotton, and corn contributing to 33%, 21%, and 12% of the total harvest acreage of the top 13 crops, respectively [72]. Interestingly, *Nomia* sp. (family Halictidae) are considered specialized pollinators of alfalfa [73,74]. The *Nomia* bees collected as part of this study were found nesting in an urban area with the closet farms being ~3 km away; therefore, although this virus is infecting some plant species within the area, further studies are needed to determine if it is infecting cultivated or non-cultivated plants. Although no mastreviruses have been previously found in Arizona, diverse geminiviruses infecting cacti have been found [75,76]. Nomiamastrel virus is one of the few mastreviruses/mastrevirus-like sequences to be identified in the Americas. The others include the dragonfly-associated mastrevirus [7], maize striate mosaic virus [77,78], sugarcane striate virus [79], switchgrass mosaic-associated virus 1 [80], and sweet potato symptomless virus 1 [57,58,81]. Although the vector of this virus remains unknown, it can be hypothesized that given the phylogenetic relationship of the CP amino acid sequence to that of other mastreviruses, it is likely vectored by a leafhopper.

Some plant-infecting viruses, especially RNA viruses, have been identified in pollen, reviewed in Fetters and Ashman [29] and Mink [82]; thus, it is not surprising that many of these viruses are detected and identified from plant pollinators. Within the context of geminiviruses, there are only a handful of reports of the identification of viruses in the genus *Begomovirus* in floral parts including pollen and seeds [83,84,85,86,87,88,89,90]. Thus, it is likely that we have sampled the apiscitlodal virus and nomiamastrel virus that are in floral tissue, including pollen, carried on the honeybee and solitary bees during pollination. Although there is a wealth of information on viruses in honeybees, there is limited information, e.g., Thomsom and Smirk [91], on solitary bees of the genus *Nomia*.

## 5. Conclusions

In summary, here we identify two previously unknown viruses that are clearly members of the plant-infecting *Geminiviridae* family from a honeybee and a solitary bee. This highlights that pollinators can be used to identify plant-infecting DNA viruses in addition to what has been demonstrated for plant-infecting RNA viruses [29] for virus surveillance in ecosystems. Nonetheless, the plant hosts of these two viruses still need to be identified coupled with their insect vectors to address the distribution, prevalence, and impact of these viruses.

## Data Availability

The genomes of nomiamastrel virus and apiscitlodal virus have been deposited in GenBank under accession #s PP467584 and PP467585.

## Supplementary Materials

The following supporting information can be downloaded at: https://www.mdpi.com/article/10.3390/v16040602/s1, Data S1: Pairwise identities of the genomes and proteins of citlodaviruses; Data S2: Pairwise identities of the genomes and proteins of mastreviruses and nomiamastrel virus.
